# Robotic surgery for esophageal cancer: Merits and demerits

**DOI:** 10.1002/ags3.12028

**Published:** 2017-08-14

**Authors:** Yasuyuki Seto, Kazuhiko Mori, Susumu Aikou

**Affiliations:** ^1^ Department of Gastrointestinal Surgery The University of Tokyo Hospital Tokyo Japan; ^2^ Department of Surgery Mitsui Memorial Hospital Tokyo Japan

**Keywords:** clinical‐malignant, esophageal cancer, esophagus, robotic surgery

## Abstract

Since the introduction of robotic systems in esophageal surgery in 2000, the number of robotic esophagectomies has been gradually increasing worldwide, although robot‐assisted surgery is not yet regarded as standard treatment for esophageal cancer, because of its high cost and the paucity of high‐level evidence. In 2016, more than 1800 cases were operated with robot assistance. Early results with small series demonstrated feasibility and safety in both robotic transhiatal (THE) and transthoracic esophagectomies (TTE). Some studies report that the learning curve is approximately 20 cases. Following the initial series, operative results of robotic TTE have shown a tendency to improve, and oncological long‐term results are reported to be effective and acceptable: R0 resection approaches 95%, and locoregional recurrence is rare. Several recent studies have demonstrated advantages of robotic esophagectomy in lymphadenectomy compared with the thoracoscopic approach. Such technical innovations as three‐dimensional view, articulated instruments with seven degrees of movement, tremor filter etc. have the potential to outperform any conventional procedures. With the aim of preventing postoperative pulmonary complications without diminishing lymphadenectomy performance, a nontransthoracic radical esophagectomy procedure combining a video‐assisted cervical approach for the upper mediastinum and a robot‐assisted transhiatal approach for the middle and lower mediastinum, transmediastinal esophagectomy, was developed; its short‐term outcomes are promising.

Thus, the merits or demerits of robotic surgery in this field remain quite difficult to assess. However, in the near future, the merits will definitely outweigh the demerits because the esophagus is an ideal organ for a robotic approach.

## 1 INTRODUCTION

The first robotic system became available in 1998,^1^ and the da Vinci Surgical System (Intuitive Surgical, Inc., Sunnyvale, CA, USA) was approved by the United States Food and Drug Administration in 2000.^2^ Robotic surgery for esophageal disease using da Vinci was started in 2000. Hashizume et al.^3^ carried out extraction of an esophageal submucosal tumor under robotic assistance on 1 December 2000 at Kyushu University, Fukuoka, Japan; the same group carried out robot‐assisted esophagectomy for esophageal carcinoma on 15 May 2001 (personal communication). The first case of robot‐assisted esophagectomy for esophageal carcinoma reported in the literature was carried out by Horgan et al.^4^ in September 2001 at the University of Illinois, Chicago. Horgan's procedure was a transhiatal esophagectomy (THE). Robotic transthoracic radical esophagolymphadenectomy (a type of TTE) was first done in November 2002 at the University of Iowa Hospital, Iowa City, and reported by Kernstine et al.^5^

Initially, the potential advantages of this technological innovation were thought to allow surgeons to carry out more precise and safer, more minimally invasive procedures, compared with conventional laparoscopic procedures.^6^ Also, the esophagus was regarded as an ideal organ for a robotic approach,^7^ because the esophagus is anatomically located in a limited and narrow space, the mediastinum, behind such vital organs as the heart and the trachea.

**Figure 1:**
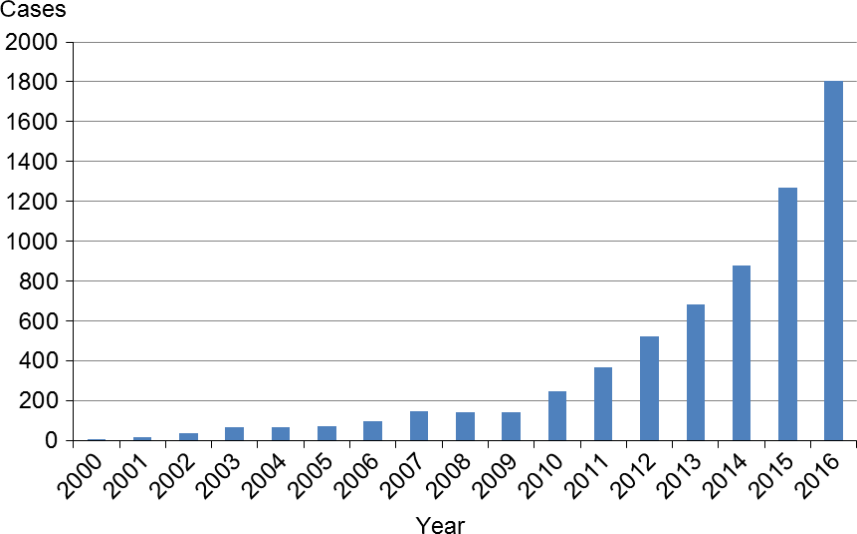
Growth in the number of robotic surgery procedures worldwide. © Intuitive Surgical, Inc.

Figure 1 shows growth in the number of robotic esophagectomy procedures using the da Vinci system around the world. Although the numbers are gradually increasing worldwide, robotic esophagectomy for esophageal cancer is not yet regarded as a standard procedure, or as superior for treatment of urological and gynecological malignancies because of the lack of clear benefits.^8^ Robotic surgery's advantages and disadvantages for esophageal cancer thus remain controversial. We reviewed this problem focusing on robotic surgery for esophageal carcinoma, although high‐level evidence is lacking because of the absence of any except currently ongoing randomized controlled trials.^9^

## 2 EARLY RESULTS WITH SMALL SERIES OF TRANSHIATAL ESOPHAGECTOMY

Initial small series of reports on transhiatal esophagectomy (THE) are reviewed here. A total of three such papers have been published.^10^, ^11^, ^12^ Respectively, numbers of patients were 18, 23, and 40; mean operative time, estimated blood loss, hospital stay, and lymph node yield were 279 min (231–311), 88 mL (54–100), 9.2 days (9–10), and 17 (14–20). Robotic THE was reported to be safe even after chemoradiotherapy.^12^ These reports showed high complication rates, approximately 50%; one patient (1/81, 1.2%) died from pulmonary failure after surgery. Table 1 summarizes the surgical outcomes of these series. One paper reported a high incidence (19.4%, 7/36) of incarcerated hiatal hernia after robotic THE.^13^ Indications for robotic THE were mostly adenocarcinomas located in the distal esophagus and gastroesophageal junctions.^10^, ^11^, ^12^

**Table 1:** Surgical outcomes of early results of robotic transhiatal esophagectomy (THE)

Author	No. cases	Operative time (min)	Blood loss (mL)	Hospital stay (days)	Dissected nodes	Pulmonary complications (%)	Anastomotic leakage (%)	Vocal cord palsy (%)
Galvani et al.^10^	18	267	54	10	14	11	33	5
Dunn et al.^11^	40	311	97	9	20	20	25	30
Coker et al.^12^	23	231	100	9	15	22	9	NA

NA, not available.

## 3 EARLY RESULTS WITH SMALL SERIES OF TRANSTHORACIC ESOPHAGECTOMY

A total of nine published papers reported initial cases of transthoracic esophagectomy (TTE).^14^, ^15^, ^16^, ^17^, ^18^, ^19^, ^20^, ^21^, ^22^ Numbers of patients ranged from 14 to 50, in total 224 cases; mean operative time, estimated blood loss, length of hospital stay, and lymph node yield were 466 min (210–666), 296 mL (80–950), 11.7 days (8–21), and 21.4 (18–38), respectively. Complication rates varied from 15% to 93%; perioperative mortality was 1.8% (4/224). Surgical outcomes of these series are presented in Table 2. Robot‐assisted TTE in the prone position was reported in two papers as initial series.^21^, ^22^ Feasibility and safety of intrathoracic hand‐sewn anastomosis and lymphadenectomy along the recurrent laryngeal nerves during robotic surgery were shown, as early results, in the studies of Cerfolio et al., Trugeda et al., Suda et al., and Kim et al., respectively.^23^, ^24^, ^25^, ^26^

**Table 2:** Surgical outcomes of early results of robotic transthoracic esophagectomy (TTE)

Author	No. cases	Operative time (min)	Blood loss (mL)	Hospital stay (days)	Dissected nodes	Pulmonary complications (%)	Anastomotic leakage (%)	Vocal cord palsy (%)
van Hillegersberg et al.^14^	21	450	950	18	20	48	14	14
Anderson et al.^15^	25	482	350	11	22	16	16	4
Kernstine et al.^16^	14	666	400	NA	18	21	14	14
Sarkaria et al.^17^	21	556	307	10	20	14	14	5
de la Fuente et al.^18^	50	445	146	11	20	10	4	NA
Wee et al.^19^	20	455	275	8	23	10	0	NA
Chiu et al.^20^	20	500	356	13	18	5	15	25
Puntambekar et al.^21^	32	210	80	9	20	9	6	6
Kim et al.^22^	21	410	150	21	38	0	19	29

NA, not available

## 4 LEARNING CURVE

Some papers focused on the learning curve with robotic surgery. Robotic console time with this innovative procedure was reported to be significantly reduced after the initial six patients.^22^ A significant reduction in total operative time was identified after the initial 20 or 30 cases.^27^, ^28^

## 5 AFTER THE INITIAL SERIES

To the best of our knowledge, very few or no series of robotic THE have been published following the initial small series; more results of robotic TTE, albeit few, have been published.^29^, ^30^, ^31^, ^32^ Surgeons still disagree over the relative merits of THE versus TTE. One paper reported seriously high morbidity in both groups.^33^ Another paper reported that TTE achieved a higher rate of R0 resections, a higher lymph node yield, and resulted in longer survival than THE, especially in advanced cases.^34^ Therefore, TTE is putatively more radical and therefore a more definitive treatment for esophageal cancer. The numbers of patients in reported robotic TTE groups range from 47 to 114, in total 329 cases, whose mean operative time, estimated blood loss, hospital stay, and lymph node yield were 355 min (205–450), 193 mL (35–625), 12.8 days (8–18), and 29.5 (18–44), respectively. Complication rates ranged from 19% to 45%; major pulmonary complications, recurrent laryngeal nerve palsy, and anastomotic leakages occurred; perioperative mortality was 2.7% (9/329). Table 3 summarizes the surgical outcomes of these series. Recent papers report that robotic esophagectomy is feasible for patients with a high body mass index,^35^ the elderly,^36^ and patients undergoing neoadjuvant chemoradiotherapy.^37^ Compared with initial periods, operative time and blood loss have been reduced, and the number of harvested lymph nodes has increased. Indications for robotic TTE are similar to those for conventional procedures, and tumor locations were mostly the middle esophagus, the lower esophagus, or the gastroesophageal junctions.^29^, ^30^, ^31^, ^32^ A potential advantage of real‐time perfusion assessment using indocyanine green and software built into the robotic console was recently reported:^38^ prevention of anastomotic leakage with allegedly easier detection of poorly perfused tissues at the anastomotic site.

**Table 3:** Surgical outcomes of robotic transthoracic esophagectomy (TTE) after initial series

Author	No. cases	Operative time (min)	Blood loss (mL)	Hospital stay (days)	Dissected nodes	Pulmonary complications (%)	Anastomotic leakage (%)	Vocal cord palsy (%)
Boone et al.^29^	47	450	625	18	29	45	21	19
Puntambekar et al.^30^	83	205	87	10	18	1	4	2
Cerfolio et al.^31^	85	360	35	8	22	7	4	NA
Park et al.^32^	114	420	209	16	44	10	15	26

NA, not available

## 6 ONCOLOGICAL LONG‐TERM RESULTS

More than 15 years have passed since robotic surgery began to be used in esophageal cancer treatment, and several papers have reported oncological long‐term results. The Utrecht group, one of the pioneers in this field, reported that, based on 108 cases, their radical resection (R0) rate was 95%, 5‐year overall survival (OS) was 42%, and locoregional recurrence was only 6%.^39^ The Yonsei group, another pioneer, also reported R0 and 3‐year OS rates of 95.7% and 85%, respectively.^40^ In their series, 3‐year OS was 77.8% even in stage IIIA disease. Both groups concluded that robotic TTE is oncologically effective and acceptable with a high R0 rate and adequate lymphadenectomy.

## 7 COMPARISON WITH CONVENTIONAL PROCEDURES

One experimental study showed that the robot‐assisted thoracic approach was associated with improved intraoperative cardiopulmonary function and less stress compared with the open thoracic approach.^41^ The study used 12 pigs and evaluated hemodynamics (central venous pressure, pulmonary vascular resistance, cardiac output, and blood gas values), substance P and cortisol levels. One retrospective study reported that the clinical incidence of postoperative delirium was significantly decreased after robotic TTE compared with open transthoracic esophagectomy.^42^

In comparisons of robotic to thoracoscopic approaches (ie minimally invasive esophagectomy), the first such paper published failed to show any clear advantages.^43^ However, it consisted of small series (11 and 26 cases) studied from 2008 to 2009. Several subsequent papers have shown the advantages of robotic procedures in lymphadenectomy compared with the thoracoscopic approach. Suda et al.^25^ reported that robotic assistance significantly reduced the incidence of recurrent laryngeal nerve palsy and hoarseness. Park et al.^44^ reported that the total number of dissected lymph nodes was significantly greater in the robotic than in the thoracoscopic groups, especially in the upper mediastinum and abdomen.

## 8 REVIEW ARTICLES

Several published review articles have focused on robotic esophagectomy.^8^, ^45^, ^46^, ^47^ All of them acknowledge the technical superiority of robotic surgery (ie a three‐dimensional view with up to 10‐fold magnification, articulated instruments with seven degrees of movement, natural hand‐eye coordination axis, and tremor filter). The longer operative time was pointed out as a disadvantage. The most important concern is that high‐level evidence of robotic esophagectomy's superiority is lacking, despite technical, oncological, and safety advantages over conventional procedures. One reason is that no randomized controlled trial of sufficient size has been conducted to show any clear benefit. Another problem is cost. Some benefit must be shown to outweigh the higher cost. The combination of fluorescence, overlay, or other advanced diagnostic imaging with robotic procedures has additional potential benefits. To conclude, robotically assisted meticulously executed procedures are expected to reduce the development of complications and improve the radicality of lymphadenectomy, which will translate into good short‐ and long‐term outcomes.

## 9 NOVEL PROCEDURES

**Figure 2:**
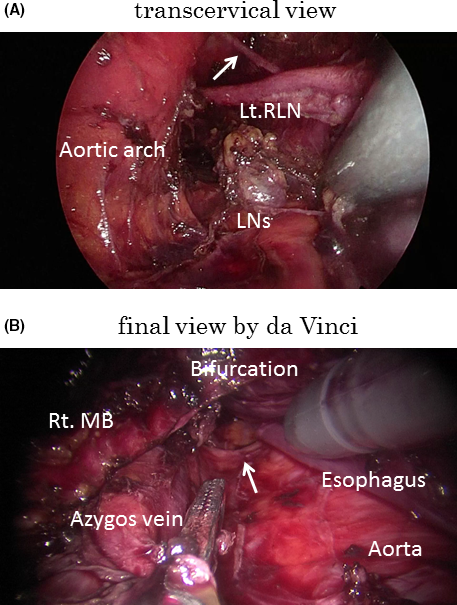
(A) Transcervical view. RLN, recurrent laryngeal nerve. Arrow shows communicating branch of RLN. (B) Final view by da Vinci Surgical System (Intuitive Surgical, Inc., Sunnyvale, CA, USA). MB, main bronchus. Arrow shows right bronchial artery

**Figure 3:**
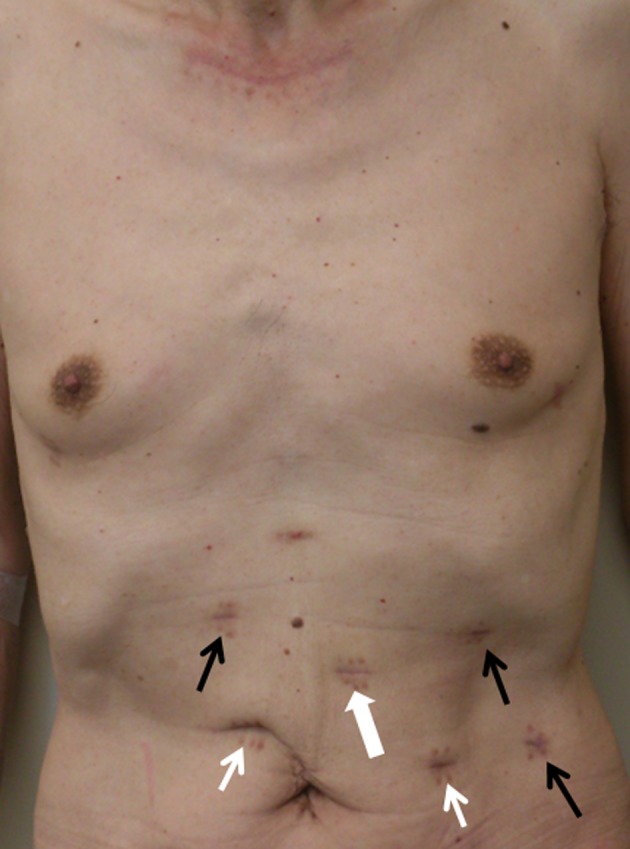
Patient who underwent transmediastinal esophagectomy. Black arrows, ports for robotic arms; bold white arrow, port for robotic camera; white arrows, ports for assistance

With the aim of averting postoperative pulmonary complications without diminishing lymphadenectomy (ie aiming at equivalence to the transthoracic approach), a nontransthoracic radical esophagectomy procedure has been developed which combines a video‐assisted cervical approach for the upper mediastinum (Figure 2A) and a robot‐assisted transhiatal approach for the middle (Figure 2B) and lower mediastinum.^48^, ^49^ The patient lies in the supine position during the operation. Neither double‐lumen intubation nor insufflation of carbon dioxide collapsing the lung, nor any change in the patient's position is necessary. Indications for the procedure were T1‐3 N0‐1 M0 thoracic esophageal cancer and no suspicion of invasion to adjacent organs. To date, 66 patients have undergone this transmediastinal esophagectomy (TME) at the University of Tokyo Hospital. No postoperative pneumonia occurred among them and oncological equivalence (ie in terms of the number of harvested lymph nodes) to conventional transthoracic surgery was confirmed. The dissection of the middle mediastinum, subcarinal, and main bronchus lymph nodes is the most important advantage of robot assistance in this procedure (cf video; this is a no‐cut edition and played at two times normal speed). An ongoing study investigating quality of life after surgery shows better results from the TME group compared with the conventional transthoracic approach group (S. Yoshimura, K. Mori, Y. Yamagata, S. Aikou, K. Yagi, M. Nishida, H. Yamashita, S. Nomura, Y. Seto, submitted). Less pain was observed after TME (Figure 3). This procedure has the potential to become a surgical option with radicality and true minimal invasiveness by applying the advantages of robotic assistance.

## 10 COMMENTS

No‐one denies the technical innovativeness and advantages of robotic surgery, and the anatomical features of the esophagus make it an ideal organ for robotic surgery. Robotic surgery therefore has merits for esophageal cancer, but it is still not regarded as a standard procedure, as a result of the paucity of definite high‐level evidence and its unacceptably high cost. We must wait for the results of ongoing randomized controlled trials to be reported^9^ and look forward to seeing competition leading to lower costs. Meanwhile, continuous endeavors to identify and develop additional areas of progress in the technology such as epochal imaging systems or TME applying the strong points of robots are crucial for academic surgeons pioneering the use of robotic systems.

## DISCLOSURE

Conflict of Interest: Authors declare no conflicts of interest for this article.
